# Behavioral alterations in antibiotic-treated mice associated with gut microbiota dysbiosis: insights from 16S rRNA and metabolomics

**DOI:** 10.3389/fnins.2025.1478304

**Published:** 2025-02-28

**Authors:** Asma Bibi, Famin Zhang, Jilong Shen, Ahmad Ud Din, Yuanhong Xu

**Affiliations:** ^1^The Key Laboratory of Microbiology and Parasitology Anhui, School of Basic Medical Sciences, The First Affiliated Hospital of Anhui Medical University, Hefei, China; ^2^Department of Clinical Laboratory Diagnostics, The First Affiliated Hospital of Anhui Medical University, Hefei, China; ^3^Department of Food, Bioprocessing and Nutrition Sciences, Plants for Human Health Institute, North Carolina State University, Kannapolis, NC, United States

**Keywords:** gut microbiota, antibiotic, gut dysbiosis, brain, metabolome, anxiety-like behavior

## Abstract

The gut and brain interact through various metabolic and signaling pathways, each of which influences mental health. Gut dysbiosis caused by antibiotics is a well-known phenomenon that has serious implications for gut microbiota-brain interactions. Although antibiotics disrupt the gut microbiota’s fundamental structure, the mechanisms that modulate the response and their impact on brain function are still unclear. It is imperative to comprehend and investigate crucial regulators and factors that play important roles. We aimed to study the effect of long-term antibiotic-induced disruption of gut microbiota, host metabolomes, and brain function and, particularly, to determine the basic interactions between them by treating the C57BL/6 mice with two different, most commonly used antibiotics, ciprofloxacin and amoxicillin. Anxiety-like behavior was confirmed by the elevated plus-maze test and open field test. Gut microbes and their metabolite profiles in fecal, serum, and brain samples were determined by 16S rRNA sequencing and untargeted metabolomics. In our study, long-term antibiotic treatment exerted anxiety-like effects. The fecal microbiota and metabolite status revealed that the top five genera found were *Lactobacillus, Bacteroides, Akkermansia, Ruminococcus_gnavus_*group, and unclassified *norank_f_Muribaculaceae*. The concentration of serotonin, L-Tyrosine, 5-Hydroxy-L-tryptophan, L-Glutamic acid, L-Glutamate, 5-Hydroxyindole acetic acid, and dopaminergic synapsis was comparatively low, while adenosine was high in antibiotic-treated mice. The KEGG enrichment analysis of serum and brain samples showed that amino acid metabolism pathways, such as tryptophan metabolism, threonine metabolism, serotonergic synapsis, methionine metabolism, and neuroactive ligand-receptor interaction, were significantly decreased in antibiotic-treated mice. Our study demonstrates that long-term antibiotic use induces gut dysbiosis and alters metabolic responses, leading to the dysregulation of brain signaling molecules and anxiety-like behavior. These findings highlight the complex interactions between gut microbiota and metabolic functions, providing new insights into the influence of microbial communities on gut-brain communication.

## 1 Introduction

Trillions of microbes, collectively called “gut microbiota,” colonize the digestive tract. Gastrointestinal physiology and the function of distant organs, as well as the susceptibility of the host to disease, are influenced by this extensive microbial community residing in the colon (Lozupone et al., 2012). Due to their complex, dynamic, and metabolically active nature, these commensal microorganisms play an essential role in the development of immune cells (Hooper et al., 2012), physiological processes including energy harvesting (Turnbaugh et al., 2006), and gut epithelial cell homeostasis (Wells et al., 2011). Studies published recently suggested that gut microbiota may also have neuroactive properties that are linked to neurological and psychiatric disorders, which has been referred to as the microbiota-gut-brain axis (Cryan et al., 2020). In the gut-brain axis, neuronal, humoral, and immune signaling pathways are involved in gut microbiota and brain bidirectional communication (Bienenstock et al., 2015). Neuropsychiatric diseases are associated with disturbances of the gut microbiota, which regulates brain behavior and function (Morais et al., 2021). Metabolic, immune, endocrine, and neuronal pathways may all be affected by gut microbiota (Sharon et al., 2014).

Recent studies have revealed that chemical signals transported from the gastrointestinal lumen to the systemic circulation are synthesized or transformed by the microbiota. Their effects may impact the central nervous system (CNS) once they cross the blood–brain barrier (BBB) (Ursell et al., 2014; Liu et al., 2021). Alterations in gut microbiota metabolites and chronic systemic inflammation can lead to neuroinflammation and neurodegeneration (Mou et al., 2022). In addition to regulating the immune response and physiological metabolism, these metabolites play a role in maintaining the connection between the brain and gut microbiota (Wang et al., 2020). Approximately 90% of mammalian gut bacteria belong to the Bacteroidetes and Firmicutes phyla (Eckburg et al., 2005). The Firmicutes phylum has been shown to produce various neurotransmitters, including dopamine, which is related to the control of mood and emotional stability (Donthamsetti et al., 2020). A significant correlation was found between *Bacteroides* species and amino acid and lipid metabolism, like glutamate metabolism (Cervenka et al., 2017). The alteration of amino acid neurotransmitters, such as 5-hydroxytryptamine, by these bacteria may cause the onset of mental illness (Hamamah et al., 2022). Neurotransmitters, like *γ*-aminobutyric acid (GABA), are produced in large quantities by Firmicutes and Actinobacteria, which impacts host neurophysiology (Bravo et al., 2011). Firmicutes produce comparatively high levels of norepinephrine (Strandwitz, 2018), and they have an impact on behavior and cognitive processes, including learning, attention, and memory (Borodovitsyna et al., 2017). Recent studies have demonstrated that the gut microbiota actively controls these metabolic fluxes to influence either the distant central nervous system (CNS) or the local enteric nervous system (ENS) (Dinan and Cryan, 2017). Surprisingly, despite all this interest, the molecular foundations of this microbiota-gut-brain interplay remain poorly understood. It is not clear when and how microbiota initiate specific neurotransmitter production.

In the context of medicine, antibiotics are commonly prescribed (Swindells et al., 2018). The use of antibiotics is associated with dysfunctional metabolism and dysbiosis of the gut microbiota (Zhang N. et al., 2022). The symbiotic bacterial load and diversity in the gastrointestinal tract are rapidly reduced after broad-spectrum antibiotic use (Dethlefsen and Relman, 2011). Numerous opportunistic infections may arise from this disturbance as a result of diminished colonization resistance against different bacterial and fungal pathogens (Blaser, 2011; Mukherjee and Hooper, 2015). The disrupted microbiota leads to unwanted metabolite production, which influences brain health (De Luca and Shoenfeld, 2019). Moreover, several neuropsychiatric disorders have been linked to chronic dysbiosis of the microbiota, but so far, no direct evidence has ever been well documented.

This study aimed to establish whether gut dysbiosis leads to the alteration of gut microbiota-brain communication via microbial metabolites or the dysregulation of brain neuronal signaling systems. Considerable literature suggests the microbiota-gut-brain axis may be associated with mental health disorders. However, the effect of gut microbial alterations on metabolite production impairs cognitive behavior and mental health, and the mechanisms by which these effects occur remain inconclusive. First, there is a lack of knowledge about the association between gut dysbiosis and host systematic metabolomes and the long-term effects of antibiotic use on metabolite mediators. Second, little is known about the important roles that metabolites and associated metabolic pathways play in the brain’s primary cognitive processes and how disruption of these pathways might result in neuropsychiatric diseases. The diverse range of compounds produced by gut bacteria and their effects on the brain can lead to effective targeted interventions.

Most commonly used antibiotics, such as amoxicillin (a beta-lactam) and ciprofloxacin (a fluoroquinolone), were chosen for this study to explore the function of the murine microbiota in shaping brain neurochemistry and function. Two different antibiotics were used to examine how intragastric antibiotic treatment affects gut microbial flora, metabolite profiles, circulating metabolites, and their related pathways in the brain of mice. A majority of species were reduced in abundance by amoxicillin, except for a few members of the *Bacteroides* genus. Contrary to its effects on *Bacteroides*, ciprofloxacin increased Firmicutes abundance (Desbonnet et al., 2015; Farzi et al., 2018). Changing taxonomic structure results in changes in the relative abundance and metabolism of the gut microbiota (Cabral et al., 2019). A short-term administration of antibiotics, however, does not appear to cause significant metabolic changes (Yang et al., 2023). Given these considerations, disruption of gut microbiota (dysbiosis) due to long-term antibiotic use is considered a model of intrusion to investigate the causality in microbiota-dependent effects. Cognitive and anxiety-like behaviors were examined using the elevated plus-maze test (EMPT) and open-field test (OFT). To characterize the taxonomic profile of the gut microbiota, we used 16S rRNA gene sequencing of fecal samples to study both community-level and single-species variation across the groups. We used untargeted metabolomics analyses to examine the chemical profiles of C57BL/6 mice feces samples, serum, and brain tissues to identify the metabolic responses in the gut, serum, and brain of three different research groups (Control, Amoxicillin, Ciprofloxacin group). Hence, prolonged use of broad-spectrum antibiotics disrupts the gut microbiota-brain relationship and the mental health of the host. These results will provide a foundation for understanding the clinical application of gut microbiota in human health and mental disorders.

## 2 Materials and methods

### 2.1 Mice and antibiotic treatment

Male C57BL/6 mice (6 weeks old) were obtained and domesticated at Anhui Medical University for 1 week for assimilation. For each experiment, mice were housed in social housing conditions and fed the same diet. Subsequently, a random selection of mice was made into three groups (*n* = 7 per treatment group): the control group (*n* = 7), the amoxicillin-treated group (Amx) (*n* = 7), and the ciprofloxacin-treated group (Cip) (*n* = 7). The mice were grown in individually ventilated cages (IVC) and were kept under specific pathogen-free (SPF) settings under the following conditions: 22°C, 40–70% humidity, and a 12:12 h light/dark cycle.

### 2.2 Ethics statement

Anhui Medical University’s experimental animal ethics committee approved the animal study (permit number 20,180,016), which included guidelines for the procedures used on the animals. Regulatory and institutional requirements were followed when conducting the study.

### 2.3 Antibiotics

The antibiotics ciprofloxacin (CAS 86393–32-0) and amoxicillin (CAS 61336–70-7) were purchased from Solarbio Beijing and Macklin (Shanghai), China, respectively. Antibiotics were dissolved in PBS (pH 6.98–7.14) and were administered orally daily for 3 weeks. Dosages were based on those used in previous mouse studies (Hertz et al., 2020; Stavroulaki et al., 2021). Control mice received PBS only. Each day, the mice were weighed before the oral administration of the antibiotics, and the volume was adjusted accordingly. We used the Lab Rat and Mouse Maintenance Diet (Xietong, China)^1^ when raising mice, which is designed as the standard diet for the maintenance phase of laboratory rats and mice. The main ingredients in the feed included corn, wheat, fish meal, chicken meat meal, sodium chloride, etc., which are rich in essential amino acids and vitamins and are sufficient to meet the needs of experimental mice. Autoclaved water and food were allowed ad libitum.

### 2.4 Behavioral tests

To assess the anxiety-like behavior of mice, the OFT and EPMT were conducted during the third week of antibiotic administration (Shentu et al., 2024). The same experimental settings were used in all the experiments. A minimum of 1 h was provided for the mice to acclimatize to the testing environment before each test. Following each experiment, the equipment was thoroughly rinsed with 75% alcohol to remove mouse feces and odors, and no further experiments were conducted until the ethanol had completely evaporated. For tracking, recording, and evaluating each mouse’s ambulatory trajectory, we used Pan Lab Technology for Bioresearch of Barcelona, Spain (version 3.0).

### 2.5 Open-field test

The OFT was conducted in a room measuring 45 cm × 45 cm × 45 cm with black walls and white floors. The center of the chamber was determined as a 10-cm zone between each wall. For 5 min, the mice were placed down gently in the middle of the field and allowed to locomote about. There were fewer entries in the central region, and less time was spent there, indicating higher levels of anxiety.

### 2.6 Elevated plus-maze test

This test involved the mice walking through a cross maze with 2 open arms (30 cm length x 6 cm width) and 2 closed arms (6 cm width × 30 cm length × 15 cm height). During the experiment, the mice were placed down facing an open arm in the center of the maze and allowed to navigate freely for 5 min. Anxiety levels were determined by the number of entries and duration of the maze.

### 2.7 Sample collection

All mice were euthanized after 3 weeks, and fecal, serum, and brain tissue samples were collected for targeted analysis of relevance to the gut-microbiota axis. Fecal samples were used for 16S rRNA amplicon sequencing to analyze the microbiota and quantify microbial metabolites. Serum and brain samples were used for untargeted metabolome analyses. Fecal samples were collected after defecation on sterilized paper within 24 h of the last administration (Yang et al., 2023). Following the protocols described previously (Zhou et al., 2022), blood samples were collected after anesthetizing the mice, and serum was isolated via centrifugation and stored for further analysis. Following the protocols by Marquardt et al. (2018), immediately post-sacrifice, the brain was isolated using ultra-fine forceps after perfusion with saline. The brain was collected as described previously (Dyar et al., 2018) and maintained on ice. The brain was then immediately cryopreserved in liquid nitrogen and then stored at −80°C until further use.

### 2.8 Bacterial DNA extraction and amplification

A fecal sample of 200 mg from each mouse was used for 16S rRNA analysis. From the mice feces for the extraction of total bacterial DNA, we used the E.Z.N.A.^®^ Soil DNA Kit (Omega Bio-Tek, Norcross, GA, United Sates). DNA quality and concentration were assessed using a NanoDrop 2000 spectrophotometer (Thermo Fisher Scientific; Wilmington, NC, United Sates). Amplification of 16S rRNA genes was carried out using bacterial primers 338F (5′-ACTCCTACGGGAGGCAGCAG-3′) and 806 R (5′-GGACTACHVGGGTWTCTAAT-3′), which span the hypervariable regions of V3-V4 on a Bio-Rad T100 system (Bio-Rad, Hercules, CA, United Sates). PCR reactions for each sample were performed in triplicate in 20 μL reaction solutions containing 0.4 μL of 5× FastPfu Buffer, 2 μL of 2.5 mM dNTPs, 0.8 μL of each primer (5 μM), 0.4 μL of FastPfu polymerase, and 10 ng of template DNA. The following thermal cycler program was used for amplification: 3 min at 95°C, 27 cycles of 30 s at 95°C, 30 s at 55°C, and 45 s at 72°C, and a final extension at 72°C for 10 min. The next step was the sequencing of purified amplicons using an Illumina MiSeq platform (Illumina, San Diego, CA, United Sates) using the standard protocols of Majorbio Bio-Pharm Technology Co. Ltd., Shanghai, China.

### 2.9 16S rRNA gene sequencing analysis

To analyze and screen the quality, raw sequence reads were filtered and merged using Trimmomatic and FLASH software. Using the UPARSE pipeline (version 7.1), the OTUs were clustered after chimeric sequences were removed. The RDP Classifier^2^ was used to analyze all OTU representative sequences from the 16S rRNA gene database in Sliva 138 within the 0.7 confidence threshold. To conduct the correlation analysis, Omic Studio was used.^3^ For each library, the alpha diversity index, community diversity (Shannon and Simpson), and community richness (Ace and Chao) were calculated using Mothur v1.30.1 with OTU information. In addition, we used Mothur data to generate a rarefaction curve and species accumulation plots to confirm the sequencing depth (Schloss et al., 2009). The similarity among the samples was analyzed by PERMANOVA and principal coordinate analysis (PCoA) based on Bray–Curtis dissimilarity using the Vegan v2.5–3 package. The permutational multivariate analysis of variance (PERMANOVA) confirmed the difference using the Majorbio database. To identify significant taxa (phylum to genera) of bacteria among the different groups, we used linear discriminant analysis (LDA score > 2 was employed and *p* < 0.05 was considered significant) effect size (LEfSe).^4^ Raw data from the entire sequencing have been uploaded to the NCBI Sequence Read Archive (SRA) under accession number PRJNA1063454.

### 2.10 Metagenomics sequencing analysis

To further study the functional ability of the microbiome, we randomly selected fecal samples from each group for metagenomic sequencing analysis on an Illumina sequencing HiSeq platform (Illumina, San Diego, CA, United States) using the standard protocols of Majorbio Bio pharm Co., Ltd., Shanghai, China. After sequencing, the raw reads were cleaned with Readfq (version 8), the data were filtered using Bowtie (version 2.2.4), and the data were subjected to a BLAST search against the host database. The metagenome was then assembled using a single or mixed assembly. Single-sample assembly was performed using SOAP denovo (ver. 2.04) (Luo et al., 2012) for each sample. Using Bowtie 2 software, clean data from all samples were mapped to scaffolds, and unused paired-end reads were obtained. The mixed assemblies of all samples were performed using SOAP denovo and MEGAHIT (version 1.04-beta). Fragments below 500 bp in the scaftigs generated from the single or mixed assembly were filtered out for statistical analysis. Next, MetaGeneMark (v 2.10) (Zhu et al., 2010) was used to predict non-redundant (Nr) genes in scaftigs, and CD-HIT software (v 4.5.8) was used to obtain the unique initial gene catalog (Li and Godzik, 2006). Clean data for each sample were mapped to the initial gene catalog using Bowtie to obtain the number of reads and statistical abundance of each gene in each sample.

### 2.11 Metabolome analysis

The metabolomics study was conducted by Majorbio Biotech (Shanghai, China) using liquid chromatography-mass spectrometry. In brief, a 50 mg fecal sample, a 50 mg brain tissue sample, and a 100 μL serum sample were used, and methanol-based extraction was performed as per the protocols and methods established by Majorbio. After the mixture settled at −20°C, it was processed with a high-throughput tissue crusher Wonbio-96c (Shanghai Wanbo Biotechnology Co., Ltd., Shanghai, China) at 50 Hz for 6 min, followed by a vortex for 30 s and ultrasound at 40 kHz for 30 min at 5°C. The samples were placed at −20°C for 30 min to precipitate proteins. To perform LC–MS analysis, the supernatant was transferred carefully into vials after centrifugation at 13,000 × g and 4°C for 15 min. A Thermo UHPLC system was used for the separation of the metabolites, equipped with an ACQUITY BEH C18 column (100 mm × 2.1 mm i.d., 1.7 m; Waters, Milford, United States). An Electrospray Ionization source, equipped with a positive or negative ionization source, was used to collect mass spectrometric data using a Thermo UHPLC-Q Exactive Mass Spectrometer. To detect and align peaks in the raw data, UPLC-TOF/MS analyses were performed using Progenesis QI 2.3 (Nonlinear Dynamics, Waters, United States). A data matrix has been generated based on the preprocessing results, which contain retention time, mass-to-charge ratio values, and peak intensity values. A well-founded biochemical database, i.e., the Human Metabolome Database^5^ and the Metlin database,^6^ has been searched to obtain MS/MS fragment spectra and isotope ratio differences. An analysis of multivariate statistics was conducted using Bioconductor’s R package ropls (version 1.6.2)^7^ on Majorbio’s Cloud Platform^8^ (detailed in Supplementary material).

### 2.12 Correlation analysis network

We examined the effects of antibiotics on microbial species and their associated metabolites in fecal samples from mice. The data were visualized by using a multiscale network and heatmap. Pearson’s rank correlation was applied to display the parameter relations. Only correlations with Pearson’s correlation coefficient < −0.6 or > 0.6 and *p* < 0.05 (generated by Origin, version 2021b) were selected for network visualization. The network and heatmap were generated using Origin (version 2021b) and Cytoscape (version 3.6.1).

### 2.13 Microbiomic statistical analysis

Results were presented as mean ± standard error (SEM) for each group (*n* = 7). For 16S rRNA gene sequencing analysis (*n* = 7), to study the differential microbiota (95% confidence interval), the Wilcoxon rank-sum test or Kruskal-Wallis H test with Bonferroni correction was used (for two groups or multiple groups where appropriate). The similarity among the samples was analyzed by PERMANOVA and principal coordinate analysis (PCoA) based on Bray–Curtis dissimilarity using the Vegan v2.5–3 package. A Venn diagram was plotted using the Vegan package for distribution analysis and KEGG pathways among the different treated groups. The data were subjected to the Kolmogorov–Smirnov Test and Homogeneity Variances Test (K-S and H-V) to confirm normality and uniformity. The Student’s *t*-test was used to compare two groups, while one-way analysis of variance (ANOVA) with Tukey’s multiple comparison test was performed to compare multiple groups. *p*-value <0.05 was considered statistically significant. Statistical analysis of similarities (ANOSIM) carried out using the vegan package (v1.17–4) (Oksanen et al., 2013) and Circos (v0.69–3) was used to illustrate the abundance of functional genes (Kanehisa et al., 2019).

### 2.14 Metabolomics statistical analysis

The raw metabolite data were imported into the metabolomics processing software Progenesis QI (Waters Corporation) for peak identification, extraction, and alignment. A Venn diagram was plotted using the Vegan package to compare distribution, patterns, and KEGG pathways among the various treated groups. Moreover, Spearman’s correlation analysis was used to explore the correlation between the differential gut microbiome and differential metabolites (the significance threshold was FDR < 0.05). Metabolomics data were normalized and log-transformed. Metabolites with missing values >20% within each group were first excluded, and the preprocessed detail table was obtained using minima filling of all samples, sum normalization, RSD (QC) <30%, and log10 logarithmic transformation. A variable importance in projection (VIP) score of (orthogonal) partial least squares (OPLS) model was applied to rank the metabolites that best distinguished between the groups. The VIP threshold was set to one. Thus, *p*-values <0.05 and VI *p*-values >1 were chosen as common screening criteria for differential metabolites.

## 3 Results

### 3.1 Behavior test

**Figure 1:**
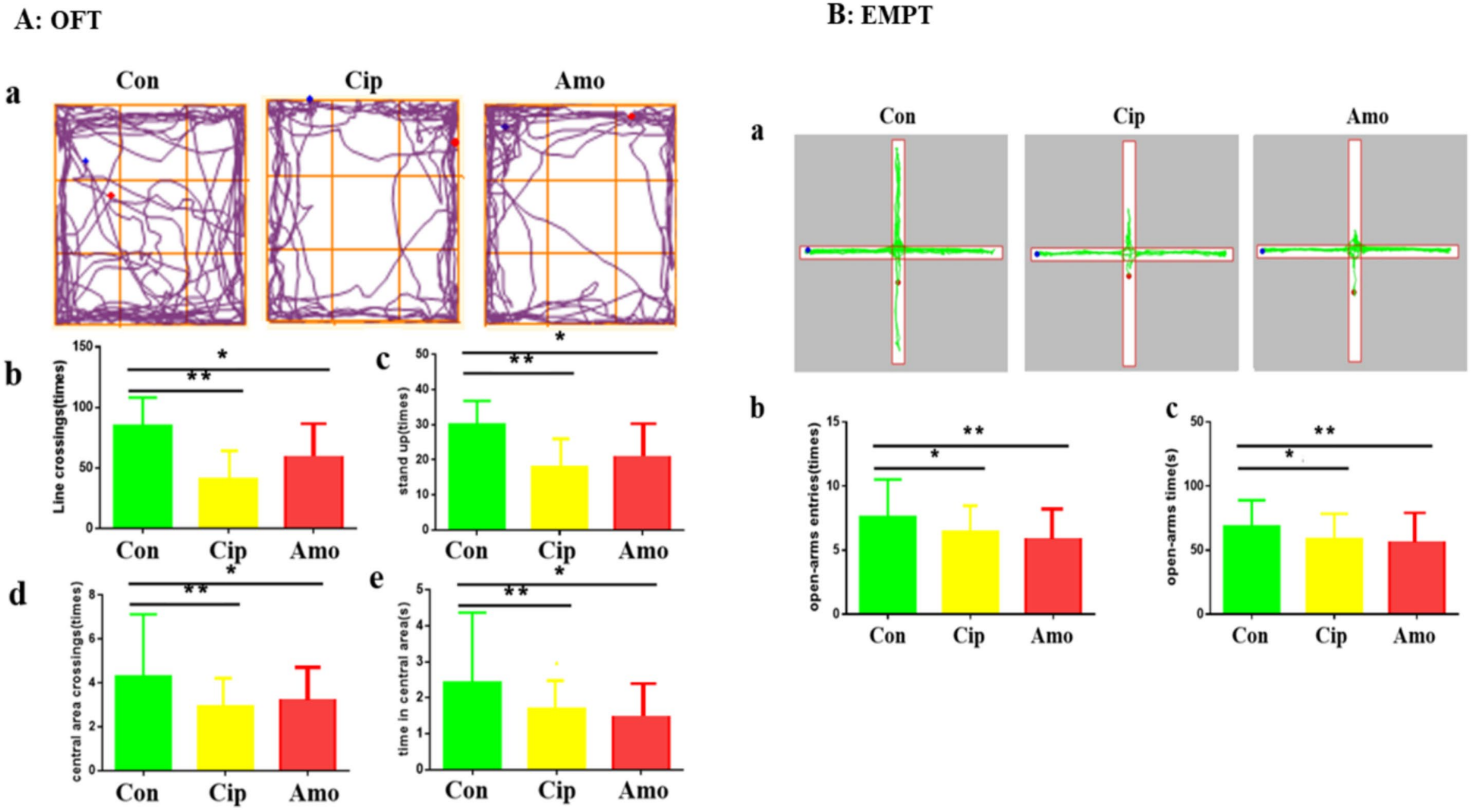
**(A)** OFT: (a) Road map of the open field experiment. (b) Number of line crossings. (c) Number of stand-ups. (d) Number of central area crossings. (e) Times in the central area. Each value represents the mean ± SEM. *p < 0.05. **(B)** EMPT: (a) Road map of the elevated plus maze. (b) Number of open-arms entries. (c) Time in open arms. Each value represents the mean ± SEM. *p*-value < 0.01 is marked as **, and *p*-value < 0.05 are marked *.

We assessed anxiety-like behavior in the control, Cip, and Amx groups. The animals showed signs of anxiety and weight loss, and the body weights of the control, Cip, and Amx groups were 20.84 ± 2.9, 17.45 ± 2.8, and 17.56 ± 3.1, respectively. The OFT and EPMT were used to analyze anxiety-like behaviors provoked by antibiotics. In OFT mice administered with ciprofloxacin and amoxicillin, the time in the center area was shorter, there were fewer entries, they exhibited a preference for the periphery or corners, and there was a decline in the range of activity compared to the control group (*p* < 0.05) (Figure 1A). The EPMT test indicated that antibiotics administered to mice markedly decreased the time spent in the open arms of the maze and the number of entries as well (*p* < 0.05). A smaller number of entries and less time spent in the open arm were observed in the treatment group compared with the control group (Figure 1B). These results indicate that long-term antibiotic use alters the microbiota and induces anxiety-like behaviors.

### 3.2 Gut microbiota composition, structure, and diversity

**Figure 2:**
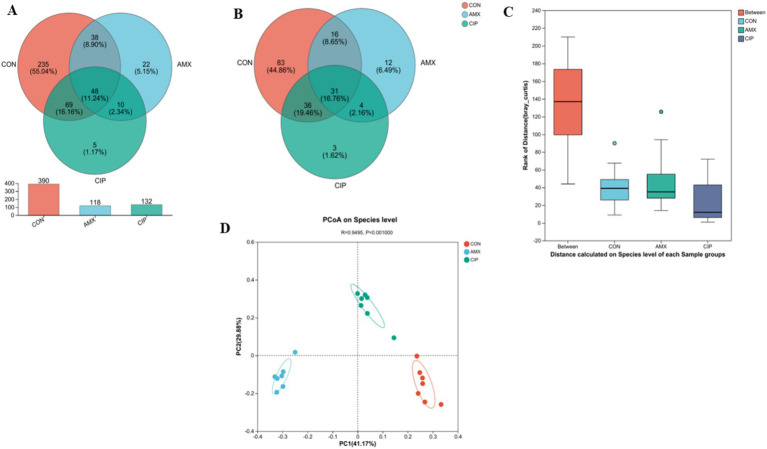
**(A)** The Venn diagram shows the number of similar and unique OTUs between different groups (Con, Amx, and Cip). **(B)** Venn Diagram species composition in groups. **(C)** Principal coordinates (PCoA) analysis of intestinal microbiota among all three groups. **(D)** The ANOSIM test showed significant differences among all three groups.

To assess microbial diversity, high-throughput sequencing was performed on 21 samples (*n* = 7), obtaining a total of 986,567 optimized sequences, 41,534,587 bases, and an average sequence length of 420 bases. A total of 640 OTUs were observed in 21 samples. The Venn diagram represented that the control group had 390 OTUs, the amoxicillin group 118, and the ciprofloxacin group 132. Of these, 48 OTUs (or 11.24% of the total) were shared across the 3 groups. The control and amoxicillin (Amx) groups shared 38 (8.90%) OTUs, the amoxicillin (Amx) and ciprofloxacin (Cip) groups shared 10 (2.34%) OTUs, and the ciprofloxacin and control groups shared 69 (16.16%) OTUs. Additionally, in the control, amoxicillin, and ciprofloxacin groups, 235, 22, and 5 OTUs were unique, respectively (Figure 2A). The Venn diagram for species composition analysis in the 3 groups showed that the control group had 166 bacterial species, amoxicillin had 63, and ciprofloxacin had 74. A total of 31 species were found in all groups; 4 species were found in amoxicillin and ciprofloxacin, 36 in ciprofloxacin and control, and 16 in amoxicillin and control. In the amoxicillin, ciprofloxacin, and control groups, there were 12, 3, and 83 unique species, respectively (Figure 2B).

### 3.3 Alpha and beta diversity indices difference in various groups

The alpha diversity estimators of bacterial species exhibit discernible differences across all three groups. The results indicate a significant increase in the species richness metrics (sobs, ace, and chao) in the control group compared with both the amoxicillin and ciprofloxacin groups (*p* < 0.05). The species diversity estimators (Shannon, Simpson, and Coverage) revealed statistically significant differences, and amoxicillin exhibited less species richness than the other two groups. Compared with the other two groups, the Ciprofloxacin group had lower species diversity. The alpha diversity of microbial species was significantly different between the amoxicillin, ciprofloxacin, and control groups (Table 1).

**Table 1:** Intestinal bacterial diversity and richness estimators in the control and treated groups.

	Mean ± SEM			*p*-value		
Estimators	Con	Amx	Cip	Amx-Cip	Con-Amx	Cip-Con
Ace	273 ± 9.76	93.17 ± 7.24	105.7 ± 4.42	> 0.1	< 0.001	< 0.001
Chao	275.8 ± 10.16	91.48 ± 8.53	104.0 ± 3.54	>0.1	< 0.001	< 0.001
Sobs	237.7 ± 11.03	85.71 ± 6.29	91.14 ± 6.25	> 0.1	< 0.001	< 0.001
Shannon	3.55 ± 0.29	3.095 ± 0.30	2.95 ± 0.12	> 0.1	< 0.01	< 0.01
Simpson	0.06 ± 0.01	0.095 ± 0.06	0.09 ± 0.01	> 0.1	< 0.01	< 0.01
Coverage	0.99 ± 0.002	0.99 ± 4.30	0.99 ± 0.01	> 0.1	< 0.001	< 0.001

Additionally, the beta diversity of all three groups showed that intestinal bacteria species were distinct based on PCoA (Bray–Curtis) (*p* = 0.001). The closer the R-value was to 1, the greater was the species difference between the groups (*R* = 0.9495) (Figure 2C). Additionally, the ANOSIM score was significantly different among all groups (Figure 2D).

### 3.4 Phylum, genus, and species level diversity difference

**Figure 3:**
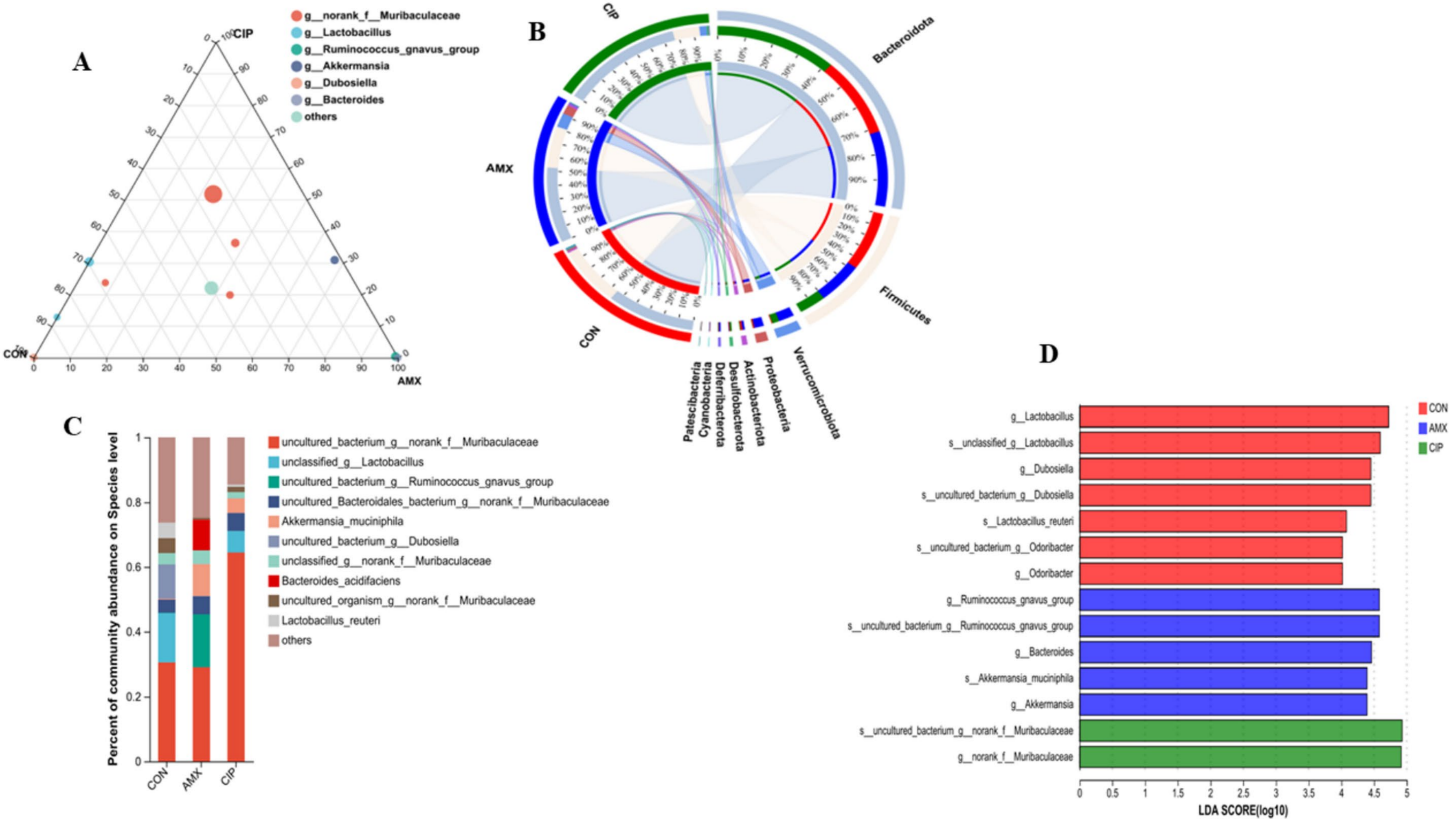
**(A)** Ternary phase diagram Visualize the composition and distribution ratio of dominant species in three different groups. The three corners represent three groups of samples. The solid circles in the figure represent species at a certain taxonomic level. The size of the circle represents the average relative abundance of species. **(B)** In the Circos sample and phylum relationship diagram, the small semi-circle (left half circle) represents the composition of phylum in the sample, the color of the outer ribbon represents which group it comes from, the color of the inner ribbon represents the phylum, and the length represents the phylum’s relative abundance in the corresponding sample; the large semicircle (right half circle) represents the distribution ratio of phylum in different samples at the taxonomic Level. **(C)** Comparison and relative abundances of intestinal bacterial species in the samples of each group. The columns of different colors represent different species, and the column length represents the proportion of the species. **(D)** The comparison and analysis of the relative differential abundance of bacterial taxa based on LDA score.

In addition, OTUs were further classified at the phylum, genus, and species taxon level. In terms of phyla, the top five phyla included Bacteroidota, Firmicutes, Verrucomicrobiota, Proteobacteria, and Actinobacteriota. The top five genera were unclassified, *Lactobacillus, Ruminococcus_gnavus_group, Akkermansia*, and *Bacteroides*. The dominating species in intestinal microbiota were *norank_f_Muribaculaceae* (unclassified) *Lactobacillus, Ruminococcus_gnavus, Akkermansia_muciniphila, Dubosiella, Bacteroides_acidifaciens, Lactobacillus_reuteri*, and other unknown less abundant species (Figure 3A). In the amoxicillin group, species abundance follows: *norank_f_Muribaculaceae* (29%) (unclassified), *Lactobacillus* (0.017%)*, Ruminococcus_gnavus* (16%)*, Akkermansia_muciniphila* (9.9%)*, Bacteroides_acidifaciens* (9.5%), and others (25%). In the ciprofloxacin group, the species abundance was *norank_f_Muribaculaceae* (64%) (unclassified), *Lactobacillus* (6.6%)*, Ruminococcus_gnavus* (0.057%)*, Akkermansia_muciniphila* (4.5%)*, Dubosiella* (0.0092%)*, Lactobacillus_reuteri* (0.70%), and others (15%). In the control group, species abundance was unclassified (31%)*, Lactobacillus* (15%)*, Ruminococcus_gnavus* (0.077%)*, Akkermansia_muciniphila* (0.24%)*, Dubosiella* (11%)*, Lactobacillus_reuteri* (4.7%), and others (26%) (Figure 3B).

Overall, this approach uses various statistical techniques in distinct groups to observe different species that are prevalent in the communities. These microorganisms could be important species linked to the emergence and progression of neuropsychiatric disorders. The amoxicillin group exhibited lower abundance of *Lactobacillus* (Firmicutes) and *Lactobacillus_reuteri* species and higher abundance of *Bacteroides_acidifaciens* (Bacteroidota) and *Akkermansia_muciniphila* (Verrucomicrobiota) compared to the control group. The ciprofloxacin group had less abundance of *Bacteroides_acidifaciens* and *Lactobacillus_reuteri* and a higher abundance of *Akkermansia_muciniphila* and unclassified than the control group. The *Lactobacillus* species abundance in the ciprofloxacin group was higher than that of the amoxicillin group, but it was lower than that of the control group (Figure 3C).

### 3.5 Differential gut microbiota analysis in various treatment groups

The LEfSe multi-level species difference discriminant analysis analyzes differential species at multiple levels. The LEfSe analysis revealed considerable microbial alteration, as represented by the bar charts (LDA ≥ 2). The LDA score coincides with the impact of species abundance. Based on the Kruskal-Wallis H test, our results highlighted that the species-level-rich bacterial taxa were Bacteroidota, unclassified, and Firmicutes in amoxicillin, ciprofloxacin, and the control group (Figure 3D).

### 3.6 Fecal metabolome composition analysis

**Figure 4:**
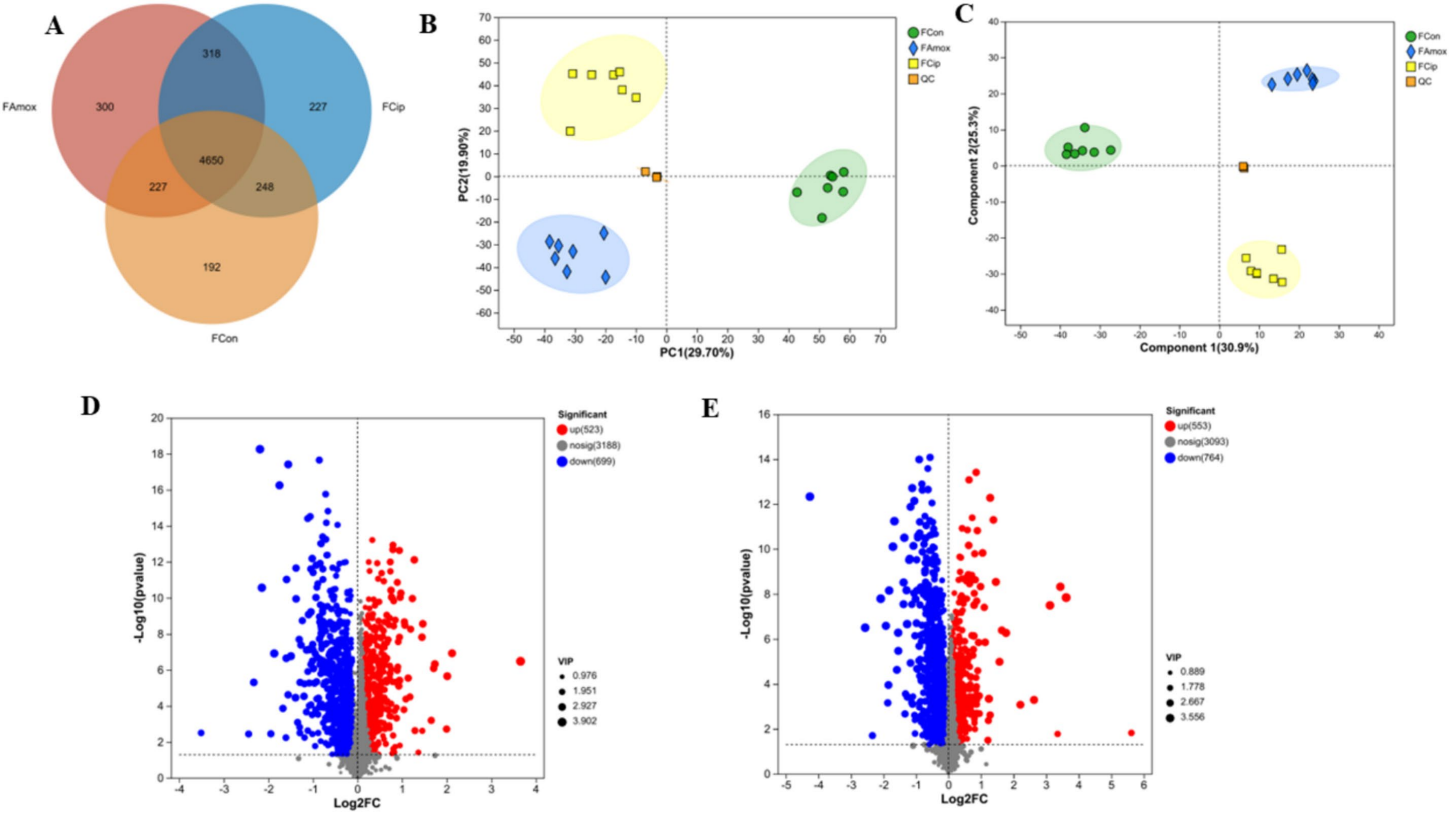
**(A)** The Venn diagram shows the number of similar and unique fecal metabolites between different (con, Amx, and Cip) groups. **(B)** PCA score plot. **(C)** PLS-DA score chart. **(D)** Amx vs. con: **(E)** Cip vs. con, volcano scatter plots of DEGs.

All three groups shared 4,650 metabolites. The control group had 192 unique metabolites, including amoxicillin 300 and ciprofloxacin 227. The ciprofloxacin and control groups shared 248 metabolites, the amoxicillin and control groups shared 227 metabolites, and the amoxicillin and ciprofloxacin groups shared 318 metabolites. The control, ciprofloxacin, and amoxicillin groups had 5,317, 5,443, and 5,495 metabolites, respectively (Figure 4A).

### 3.7 Effects of antibiotics on the metabolome in the feces of mice

**Figure 5:**
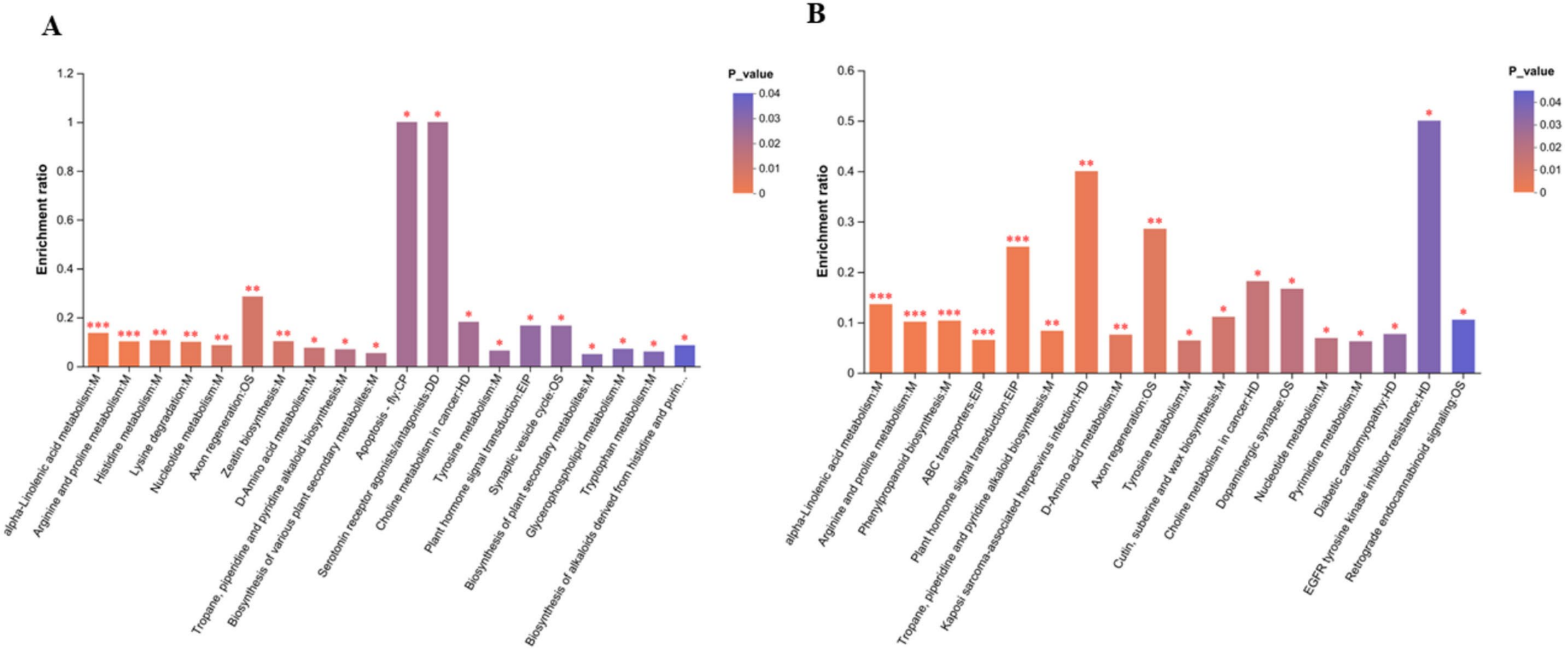
**(A)** Amx vs. con: **(B)** Cip vs. con. In the KEGG enrichment analysis graph, the abscissa represents the pathway name, and the ordinate represents the enrichment rate, which represents the ratio of the number of metabolites enriched in the pathway. *p*-value or FDR < 0.001 is marked as ***p-value or FDR < 0.01 is marked as **, and *p*-value or those with FDR < 0.05 are marked *.

An LC–MS analysis of the fecal metabolome was performed to investigate the effects of antibiotics. PCA (Principal Component Analysis) plots demonstrated a clear separation of groups in positive and negative mode (Figure 4B). In addition, metabolic patterns were estimated using PLS-DA (Partial Least Squares Discriminant Analysis) analysis, which demonstrated a strong group separation in positive and negative modes as well, suggesting that the developed approach had high stability and repeatability. In comparison to the control group, the amoxicillin and ciprofloxacin groups showed significant dysregulation of metabolites (Figure 4C). Ciprofloxacin groups showed 553 upregulated metabolites and 764 downregulated metabolites. The amoxicillin group had 523 upregulated and 699 downregulated metabolites (Figures 4D,E). The KEGG enrichment analysis revealed that antibiotic ingestion significantly altered the levels of metabolites. The amoxicillin group exhibited decreased amino acid, lysine, histidine, tyrosine, and tryptophan metabolisms. The KEGG enrichment analysis of the ciprofloxacin group revealed a reduction in the metabolism of arginine, tyrosine, dopaminergic synapsis, pyrimidine, and arginine. This decrease in metabolism is associated with symptoms such as psychosis, depression, anxiety, irritability, insomnia, and fatigue (Figures 5A,B). The findings of this study indicate a strong correlation between the use of antibiotics and the alteration of the gut metabolome produced by the gut bacteria in individuals with neuropsychosis.

### 3.8 Brain metabolome composition

**Figure 6:**
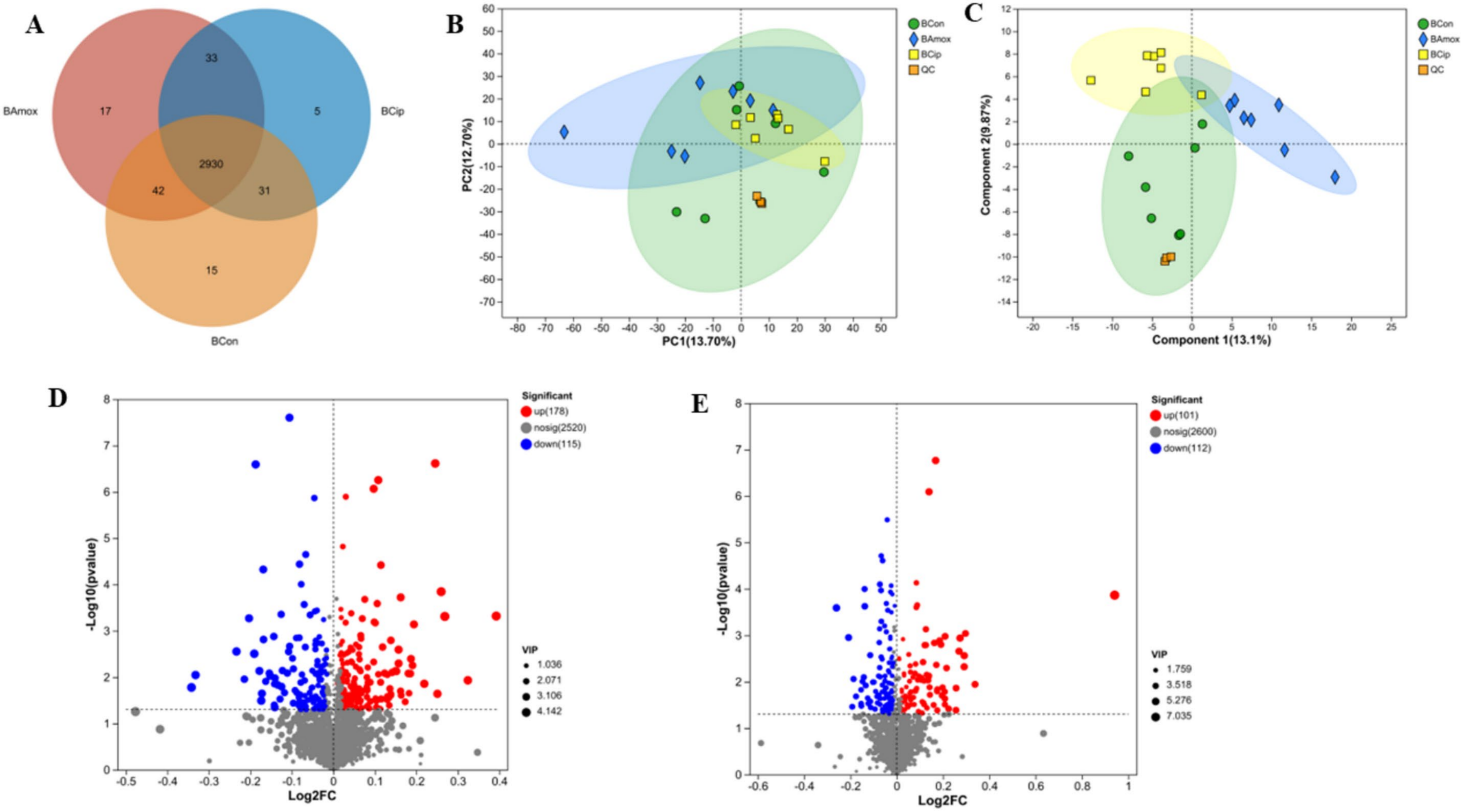
**(A)** The Venn diagram shows the number of similar and unique brain metabolites between different (con, Amx, and Cip) groups. **(B)** Principal component analysis (PCA) diagram of brain metabolites. **(C)** PLS-DA score chart. **(D)** Amx vs. con: **(E)** Cip vs. con, volcano scatter plots of brain metabolites.

In brain tissue, 2,930 metabolites were common to all 3 groups. Amoxicillin had 17 unique metabolites, ciprofloxacin had 5, and the control group had 15 unique metabolites. The control and ciprofloxacin groups shared 31 metabolites, the amoxicillin and control groups shared 42 metabolites, and the ciprofloxacin and amoxicillin groups shared 33 metabolites. The control group had 3,018 metabolites, the ciprofloxacin group had 2,999, and the amoxicillin group had 3,022 metabolites in the brain tissue samples (Figure 6A).

### 3.9 Brain metabolites analysis

**Figure 7:**
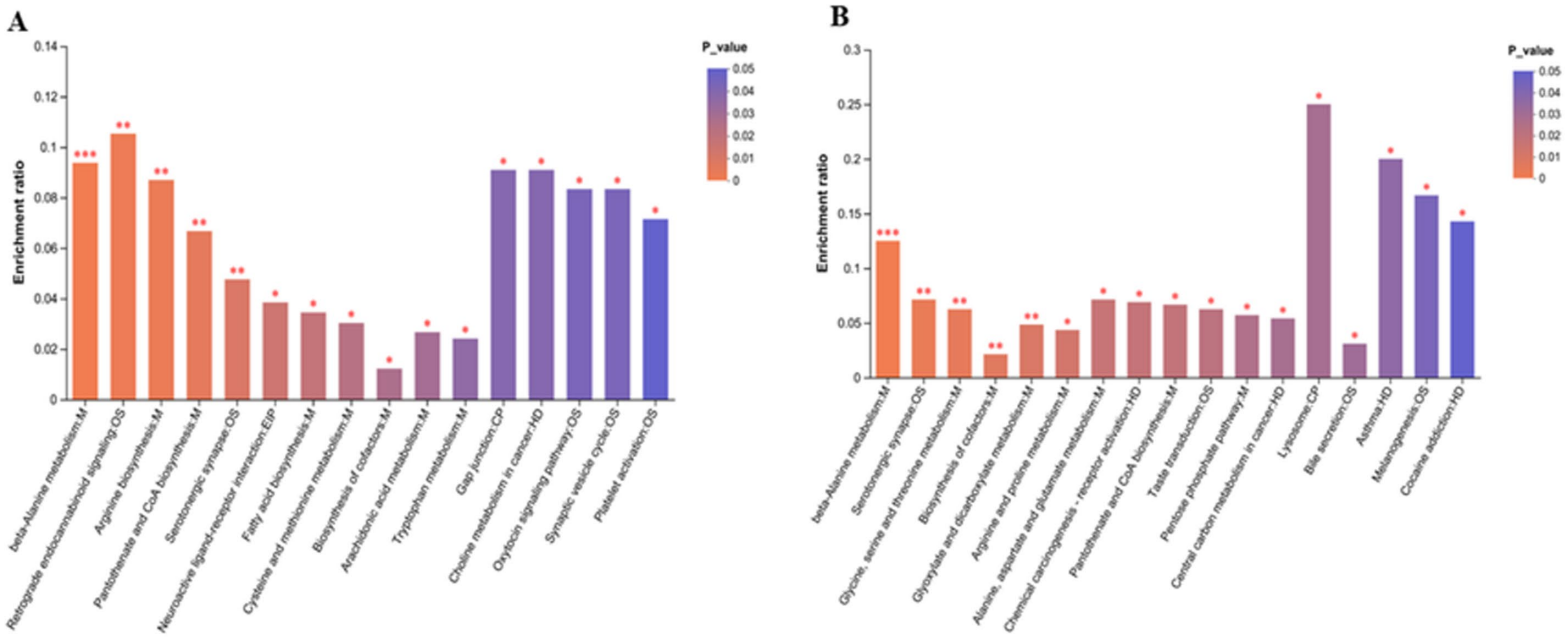
**(A)** Amx vs. con: **(B)** Cip vs. con, KEGG enrichment analysis graph of brain metabolic pathways in antibiotic-treated vs. control group. *p < 0.05, **p < 0.01, and ***p < 0.001.

After amoxicillin and ciprofloxacin exposure, the expression of brain metabolites was examined. PLS-DA and PCA provided effective differentiation between the three metabolic groups (Figures 6B,C). The volcano plots show the upregulation and downregulation of metabolites in the groups. In comparison with the control group, brain tissue samples treated with amoxicillin exhibited upregulation of 178 metabolites and downregulation of 115 metabolites (Figure 6D). In comparison with the control group, 101 metabolites were upregulated and 112 were downregulated in brain tissue samples treated with ciprofloxacin (Figure 6E). The KEGG enrichment analysis revealed the distribution of metabolites across several pathways. In comparison with the control group, significant reductions in metabolic pathways and metabolite expressions were seen in the group administered with amoxicillin, i.e., a decrease in cysteine and methionine metabolism, serotonergic synaptic transmission, amino acid metabolism, tryptophan metabolism, and neuroactive ligand-receptor interactions was observed. There was a noticeable decrease in metabolic enrichment of beta-alanine metabolism in the control vs. Amx group as well. The metabolite enrichment in glutamate metabolism, arginine and proline metabolism, and serotonergic synapsis was significantly reduced in the ciprofloxacin vs. control group (Figures 7A,B). The heat map illustrates the top 38 metabolites related to the nervous system that differ noticeably between ciprofloxacin, amoxicillin, and control (Supplementary Figure S1). By comparing the control group, amoxicillin, and ciprofloxacin groups, the antibiotic-treated group had significantly lower levels of L-glutamate, L-tyrosine, L-tryptophan, L-glutamic acid, Serotonin, and Dopamine. Results from these studies demonstrate that antibiotic stress alters the metabolic profile of brain tissue.

### 3.10 Serum metabolome composition analyses

**Figure 8:**
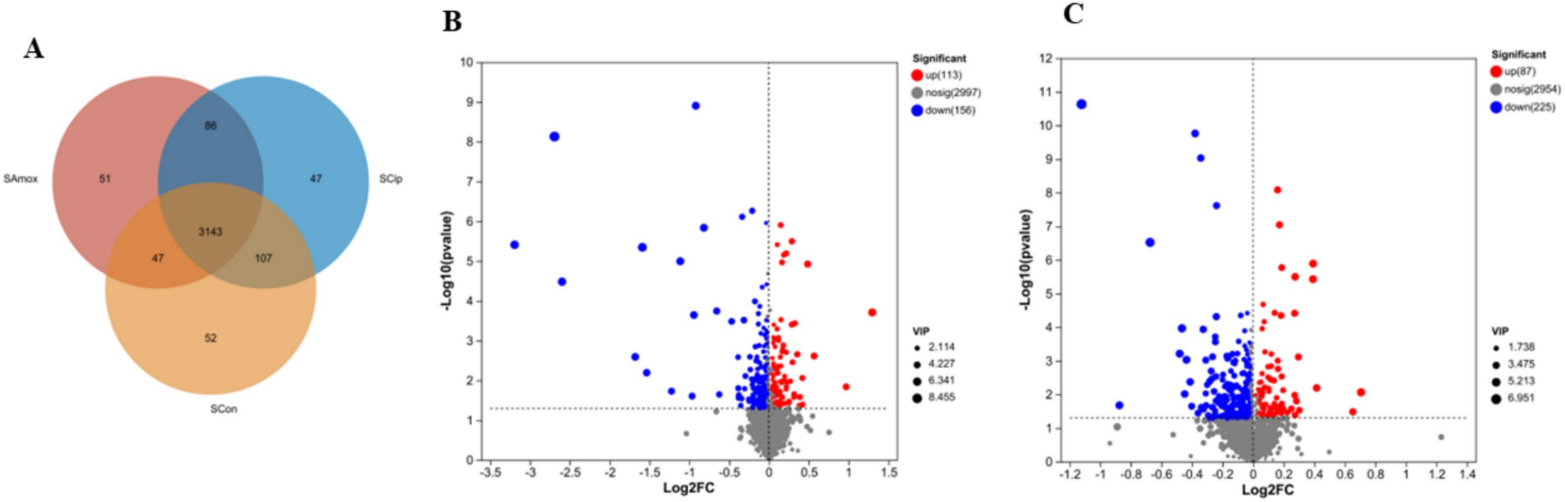
**(A)** The Venn diagram shows the number of similar and unique serum metabolites between different (con, Amx, and Cip) groups. **(B)** Amx vs. con. **(C)** Cip vs. con. Volcano scatter plots of serum metabolites.

All 3 groups shared 3,143 metabolites in the serum sample. There were 47 unique serum metabolites in the ciprofloxacin group, 51 in the amoxicillin group, and 52 in the control group. The amoxicillin and ciprofloxacin groups shared 86 serum metabolites, the control and ciprofloxacin groups shared 107, and the control and amoxicillin groups shared 47 metabolites. The amoxicillin group had a total of 3,327 serum metabolites, the ciprofloxacin group had 3,383 serum metabolites, and the control group had 3,349 serum metabolites (Figure 8A).

### 3.11 Alteration in serum metabolite profile after antibiotic treatment

After exposure to amoxicillin and ciprofloxacin, serum metabolic changes were examined. PCA analysis confirmed the robustness of the models (Supplementary Figure S2A). Significant metabolic differences were found across the groups according to PLS-DA analysis (Supplementary Figure S2B). Volcano plots showed the upregulation and downregulation of metabolites in the serum samples of the antibiotic-treated vs. control group. The serum samples from the amoxicillin vs. control group downregulated 156 and upregulated 113 metabolites (Figure 8B). In the serum samples, 87 metabolites were upregulated and 225 were downregulated in the ciprofloxacin group compared with the control group (Figure 8C). Based on KEGG enrichment analysis, the metabolite enrichment in amino acid metabolism was significantly decreased, resulting in altered pathways related to amino acids. In the serum samples of the amoxicillin-treated vs. control group, the lysine degradation pathway was notably reduced. In the ciprofloxacin vs. control groups, tryptophan metabolism, serotonergic synapses, glycine, serine, and threonine metabolism were markedly reduced (Supplementary Figures S3A,B). The heat map represents 42 significantly different metabolites among the serum samples of the three groups related to the nervous system (Supplementary Figure S3C). These changes in the amino acid pathways related to the nervous system might lead to the alteration of normal brain functions. Considering these results, we conclude that antibiotic stress induces changes in serum metabolites.

### 3.12 Antibiotic treatment correlates with gut microbiota dysbiosis and differential metabolome profiles

**Figure 9:**
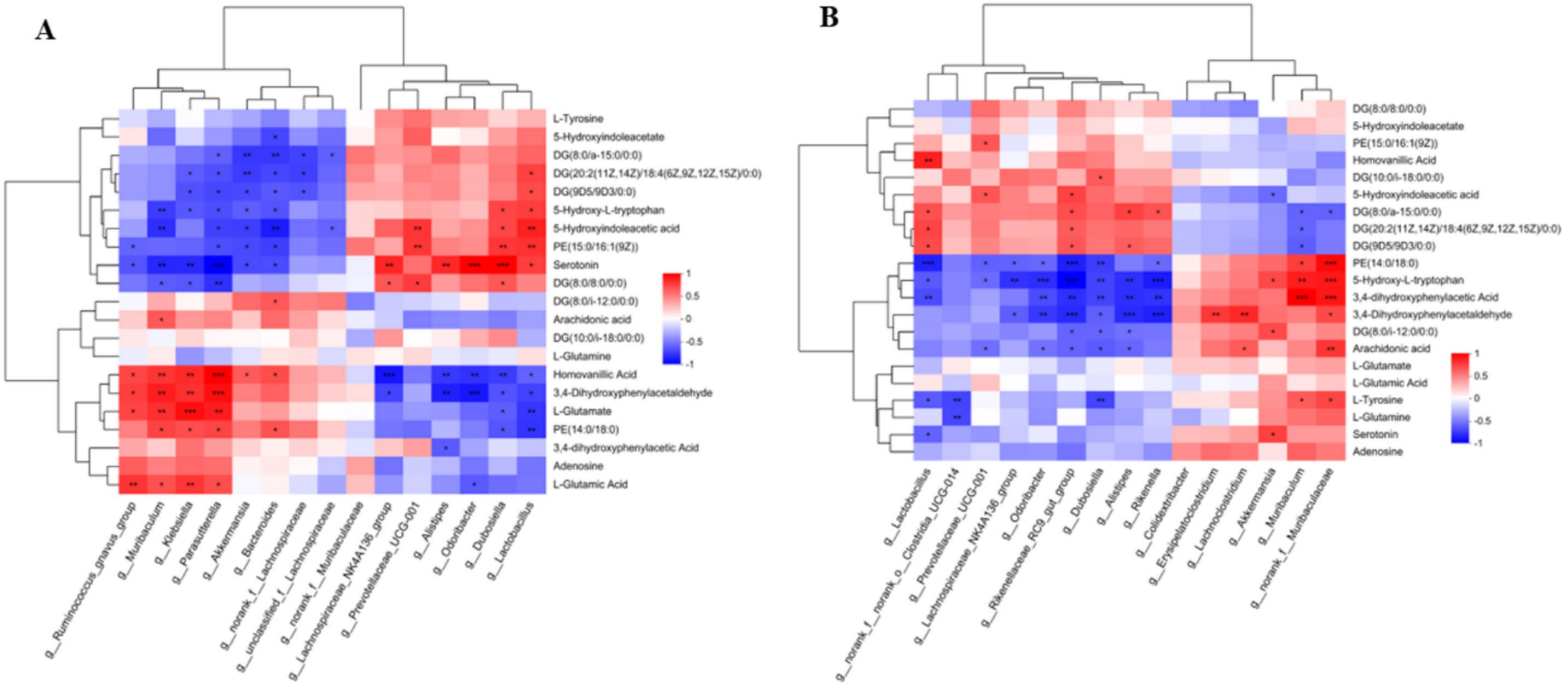
**(A)** Hierarchical clustering of correlation between gut microbiota and metabolome amoxicillin vs. control. **(B)** Hierarchical clustering of correlation between gut microbiota and metabolome ciprofloxacin vs. control.

Based on Pearson correlation analysis, the relationship between gut microbiota and differential metabolome after antibiotic treatment was examined. An analysis of 21 differential metabolites related to gut microbiota was conducted and presented as a heatmap (Figures 9A,B). Microbial alteration in the gut is linked to metabolites in the nervous system. The amoxicillin group exhibited lower abundance of the phylum Firmicutes (*Lactobacillus*) and greater abundance of the phylum Bacteroidota (*Bacteroides acidifaciens*). Compared with the amoxicillin group, the ciprofloxacin group had a lower abundance of Bacteroidota (*Bacteroides acidifaciens*) and a higher abundance of Firmicutes (*Lactobacillus*). Compared with the treatment groups, the control group had a higher abundance of *Lactobacillus* and a lower abundance of *Bacteroides acidifaciens*. The ciprofloxacin and amoxicillin groups exhibited a higher abundance of *Akkermansia_muciniphila* (Verrucomicrobiota) and a lower abundance of *Lactobacillus_reuteri* compared to the control group. A significant correlation was found between amino acid and lipid metabolism in the Bacteroidota phylum, such as glutamate and tryptophan metabolism. The phylum Firmicutes participates in dopamine production. Dopamine production was significantly reduced due to a decrease in phylum Firmicutes after antibiotic treatment. *Lactobacillus reuteri* is known to be able to produce *γ*-aminobutyric acid (GABA) in the antibiotic-treated group, and the expression of GABA activity notably decreased. The levels of L-Tyrosine, serotonin, and L-glutamic acid 5-Hydroxy-L-tryptophan, L-Glutamate, and 5-Hydroxyindole acetic acid were relatively low. In contrast to the control group, the amoxicillin and ciprofloxacin groups had higher levels of adenosine. In particular, due to a decrease in the microbial species, the levels of the short-chain fatty acids (SCFA) propionate, acetate, trimethylamine, butyrate, uracil, and adenine were significantly decreased. Several pathways of the study were strongly correlated with brain and serum metabolites in the downstream analysis. In fecal metabolites, a reduction in amino acid metabolism, including the lysine, histidine, tyrosine, tryptophan, and arginine pathways, along with pyrimidine metabolism and dopaminergic synapse function, can severely affect neurological and physiological health. Mood disorders, cognitive decline, and fatigue can be caused by impaired lysine and histidine metabolism, as well as decreased tyrosine and tryptophan metabolism. Vascular and immune dysfunction is caused by impaired arginine metabolism. The brain’s function and overall health are profoundly affected when key neurotransmitters and precursors, such as glutamate, tyrosine, tryptophan, and L-glutamic acid, are at low levels. L-glutamate and L-glutamic acid are critical excitatory neurotransmitters in the brain, and their reduction impairs cognitive function, memory, and learning. These changes in pathways related to the nervous system may lead to the altered normal brain functions. In light of these results, we conclude that antibiotic stress leads to changes in serum metabolites (Supplementary Figure S4; Supplementary Table 1). This alteration of the gut microbiota resulting from antibiotic stress disturbs the expression of metabolites.

## 4 Discussion

Recent studies have shown that the gut microbiota and the brain communicate bidirectionally and that gut dysbiosis is strongly linked to the development of psychiatric disorders, such as Alzheimer’s disease (Rieder et al., 2017). A clear understanding of the biochemical basis for such microbial effects remains elusive concerning the gut-brain axis. To efficiently develop the host body and brain functions, the colonization of gut bacteria at birth and their evolution and development after birth is more essential (Wang et al., 2022). A steady neurological state is maintained by the gut microbiota, which constantly provides appropriate signals to the brain. The consumption of antibiotics, particularly oral ones, reduces bacterial diversity and increases antibiotic resistance, gut dysbiosis, and psychiatric disorders in humans and animals. Oral antibiotic administration often has a more pronounced impact on the gut microbiota (Modi et al., 2014; Yu et al., 2020). Numerous studies have demonstrated the gut microbiota’s diversity, functional capability, and age-related changes have been extensively linked to a wide range of diseases, including localized gastroenterological problems and systemic autoimmune diseases (Vatanen et al., 2016) and diabetes (Qin et al., 2012). The fundamental mechanisms underlying changes in gut microbes and their metabolomes are, however, not well studied. Therefore, we developed a long-term antibiotic-treated mice model and conducted an investigative study of the effects of long-term antibiotic treatment on microbial communities and metabolism in the intestinal tract, as well as their effects on psychological well-being.

Accordingly, we observed in this study that mice treated with amoxicillin and ciprofloxacin experienced a change in the diversity of gut bacteria and their metabolites. However, there were differences in the type and degree of change among the groups. We found that long-term antibiotic use affected the composition of the murine gut microbiota; e.g., amoxicillin enhanced Bacteroidetes species, whereas ciprofloxacin increased Firmicutes, compared with the control. The long-term administration of these antibiotics resulted in disruption of the gut microbiota and impairment of metabolic pathways associated with cognitive dysfunction, anxiety, and depression-like behavior in mice. All animals treated with antibiotics exhibited altered flora diversity and richness, as examined by 16S rRNA gene sequencing. The amoxicillin group had a lower abundance of the phylum Firmicutes (*Lactobacillus*) and a higher abundance of Bacteroidota (*Bacteroides_acidifaciens*). The same findings were reported by Cabral et al. (2019), who found that amoxicillin significantly decreased the relative abundance of almost all species, with the notable exception of a few Bacteroides genus members (Jalili-Firoozinezhad et al., 2019). In our study, we observed a greater abundance of Firmicutes (*Lactobacillus*) in the ciprofloxacin group, consistent with the findings of Xu et al. (2020), who reported a significant abundance of Lactobacillus species in the ciprofloxacin-treated groups. This may be attributed to the inherent resistance that *Lactobacilli* possess toward ciprofloxacin, as suggested by Abriouel et al. (2015). Previous studies have indicated that the Firmicutes phylum produces various neurotransmitters, including dopamine and neurotransmitters like *γ*-aminobutyric acid (GABA), which are related to the control of mood and emotional stability (Donthamsetti et al., 2020). Our results indicate that long-term treatment with antibiotics decreases phylum Firmicutes, and hence, there was less dopamine and GABA production. Similarly, *Bacteroides* species are significantly correlated with amino acid and lipid metabolism, such as glutamate, which has a strong impact on host neurophysiology (Cervenka et al., 2017). As per our findings, there was less abundance of the phylum Bacteroidota after antibiotic treatment, which can lead to impairment of cognitive behavior. The ciprofloxacin and amoxicillin group had a higher abundance of *Akkermansia_muciniphila* (Verrucomicrobiota) compared with the control group. Previous studies have highlighted the strong correlation between severe anxiety symptoms and the high abundance of *A. muciniphila* (Zhang X. et al., 2022). However, our results indicate that the richness of *Lactobacillus reuteri* species was noticeably reduced in antibiotic-treated mice. Researchers have demonstrated that reductions in *Lactobacillus reuteri* increase stress hormone levels and decrease the expression of GABA receptors, resulting in anxiety-like behavior in animals (Mackos et al., 2013). It was enlightened by our results that alterations in gut microbial composition adversely affect metabolite expression due to long-term antibiotic stress, which can contribute to neurological disorders such as insomnia, psychosis, anxiety, and depression.

Our findings demonstrated that the changes observed in community dynamics during antibiotic treatment are the outcomes of a particular xenobiotic due to the multiplex connection between host and inter-microbial metabolism. Thus, noticeable changes could be the outcomes of indirect fluctuations reflecting host physiology or alterations in microbiota makeup. Studies have shown that broad-spectrum antibiotics induce rapid but typically brief depletion of bacterial counts and diversity within the gut microbiota (Dethlefsen and Relman, 2011). These alterations, commonly called dysbiosis, may impair colonization resistance to fungal and bacterial pathogens, resulting in opportunistic infections (Blaser, 2011; Mukherjee et al., 2022). Moreover, long-term gut microbe dysbiosis has been correlated with several chronic conditions, including asthma, inflammatory bowel disease, obesity, diabetes mellitus, autoimmune diseases, and neuropsychiatric conditions (Dickerson et al., 2017; Leong et al., 2018; De Luca and Shoenfeld, 2019).

To gain additional insight into the microbiota’s functional role in neuropsychiatric disorders by modulating the brain axis, we explored the production of related metabolites. Microbial metabolites are involved in the regulation of various metabolic pathways and can diffuse into the circulation as well (Fröhlich et al., 2016; Lee et al., 2020). Neurotransmitters are also metabolites that hamper neurological conditions (Chen et al., 2022). To understand the metabolite role, we analyzed and investigated fecal, serum, and brain tissue and correlated the alteration in microbial diversity with metabolic expression. An analysis of untargeted metabolites from fecal samples explains the upregulation and downregulation of metabolites related to gut microbiota and their enrichment in metabolic pathways. A significant difference in the metabolic status of mice treated with amoxicillin or ciprofloxacin was observed, consistent with the findings of bacterial makeup and high abundance. Different amino acid metabolisms, such as lysine, histidine metabolism, tyrosine, and tryptophan alteration or degradation, are related to mood swings, sleeping behavior (i.e., insomnia), and other behavior changes (Schopman et al., 2021). KEGG enrichment analysis in the amoxicillin group showed that amino acid metabolism, lysine degradation, histidine metabolism, tyrosine, and tryptophan metabolism were decreased. In the ciprofloxacin group, amino acid metabolism, arginine, tyrosine, dopaminergic synapsis, and pyrimidine metabolism were decreased, which is also explained well in previous scientific research studies (Puiman et al., 2013; Knoll et al., 2021).

Alterations in the levels of various chemicals circulating in the body due to gut dysbiosis may serve as signals to the brain, potentially altering its functioning (Fröhlich et al., 2016). The untargeted analysis of serum metabolomes revealed significant disruptions in the metabolism of metabolites and their related pathways. A significant decrease in tryptophan metabolism, a major mediator of depression, was observed in serum samples treated with ciprofloxacin. Caspani and Swann (2019) proposed that these circulating metabolites act as messengers between gut dysbiosis and the brain. The findings of this study contribute to the search for specialized metabolites of the gut microbiota affecting brain function in both health and disease.

Through the analysis of brain metabolites via untargeted metabolome analysis, we discovered that their related functions had changed significantly. Compared with the control, 178 metabolites were upregulated in the brain tissues of the amoxicillin group, whereas 115 metabolites were downregulated. A comparison of the ciprofloxacin and control groups showed that 101 metabolites were upregulated and 112 were downregulated. Metabolite enrichment and related pathways were seriously disrupted. In the amoxicillin group, the levels of cysteine and methionine metabolism, tryptophan metabolism, serotonergic synaptic transmission, neuroactive ligand-receptor interactions, and other amino acid metabolism pathways were markedly reduced, similar to the findings in Ma et al. (2023) used antibiotics to explore metabolic reprogramming in the intestine and concluded that downregulation of microbial metabolic events may serve as biomarkers that can modulate body homeostasis. Ciprofloxacin considerably decreased the metabolism of glutamate, arginine, proline, aspartate, beta-alanine, and serotonergic synaptic transmission. Similarly, Lubitz and Wendisch (2016) examined the metabolic changes in response to ciprofloxacin stress and highlighted that a decline in glutamate metabolism results in neurological disorders. Depression-like behavior may result from this alteration in tryptophan metabolism (Songtachalert et al., 2018). The serotonin and kynurenine pathways are two pathways through which tryptophan is metabolized, and the formation of kynurenine metabolites, neuroactive serotonin, and melatonin occurs. These pathways help in maintaining healthy homeostasis and brain functions (Richler and Gauthier, 2014).

Consistent with prior findings (Ozden et al., 2020), the current KEGG pathway analysis showed that alterations in the metabolic pathways are linked with mental illness, depression, anxiety, moodiness, trouble sleeping, tiredness, and psychosis in the antibiotic treatment groups. Zhang N. et al. (2022) and Zhang X. et al. (2022) reported that metabolic changes occur more slowly than microbial changes; our results also aligned with this study, suggesting that the metabolic alteration may have been caused by long-term antibiotic stress (Zhang N. et al., 2022). Previous studies have indicated that a decrease in the levels of the neurotransmitters serotonin and norepinephrine may lead to depression, anxiety, chronic pain, and difficulty sleeping (Zangrossi and Graeff, 2014), which is also observed in our research. Further, our results showed that due to the long-term use of antibiotics, there is an alteration in the gut microbiota that leads to a decrease in synaptic connections and less dopamine production. Likewise, previous research has shown that dopamine neurotransmitter deprivation may cause insomnia, impaired cognitive behavior, and stress (Zarrindast and Khakpai, 2015). Similarly, in rats, the amino acid alanine can modulate anxiety-like behaviors (Hoffman et al., 2019). According to our research, alanine metabolism is significantly reduced in the brain after antibiotic treatment, which is the major factor causing anxiety-like behavior. Additionally, previous studies have highlighted that the amino acid glycine sends slowing signals to the brain and sends excitatory signals to other cell types to maintain the right balance and improve mood (Li et al., 2019). However, in our study, glycine metabolism was remarkably reduced in the antibiotic-treated groups, which is likely to cause anxiety and other mood disorders. Scientific studies have shown that glutamate is one of the most important neurotransmitters in the brain (Balkhi et al., 2020); in GABAergic neurons, glutamate prepares for the synthesis of inhibitory gamma-aminobutyric acid (GABA). Neuropsychiatric disorders are associated with glutamate metabolism deficits (McGrath et al., 2022). In our research study, we found that glutamate metabolism-associated pathways were significantly decreased with the antibiotic regime, and GABA activity was reduced, which is associated with the development of anxiety and mood disorders.

## 5 Conclusion and future perspectives

The altered gut microbiota and circulating metabolites disturb brain functions and induce impaired cognitive behavior, anxiety, depression, and other neuropsychiatric disorders by modulating the gut-brain axis. These findings will uncover a theoretical framework related to a complex network that incorporates the different connections between gut microbiota and metabolites, which have a comprehensive effect on brain functions after long-term antibiotic treatment. New therapies, especially fecal microbiota transplants, show promise in preventing or reversing these adverse effects. Nevertheless, to reduce the dual threat of resistance- and antibiotic-induced dysbiosis, strategies need to be developed to improve drug efficacy while also reducing treatment-associated disruption of the microbiota. To achieve this, we must improve our understanding of antibiotic activity *in vivo* and identify the strategies bacteria use to survive treatment. Considering the aforementioned elements collectively, the long-term use of broad-spectrum antibiotics appears to induce significant alterations in the composition of metabolites and the gut microbiota, in addition to pathways that might be associated with brain-gut communication. By integrating the various interactions between gut microbiota metabolic functions that contribute to severe mental illness and neuropsychiatric disorders via gut microbiota-related metabolism, our study offers a novel perspective on a complex network. In this study, we investigated the microbiota-gut-brain axis and highlighted that antibiotic-induced gut dysbiosis can be used as a model system to examine causality between gut microbiota and the brain.

Taking all together, we conclude that long-term intragastric antibiotic treatment is directly linked to neuropsychiatric diseases, such as anxiety and depression, by adversely affecting the gut microbial community, metabolite profiles in fecal samples, circulating metabolites in serum, metabolic profile expression, and related brain pathways. The microbiota-gut-brain axis is highlighted by variations in the structure and function of gut microbes, which may also contribute to interindividual variance in the metabolic responses for microbial activation in the host. Alteration of metabolic status and its effect on neurosignaling pathways could only be a way to address the concerns and find a related mechanism that emphasizes the impact of antibiotics on the gut-brain axis, mental health, and cognitive impairment. Hence, these data support that gut microbiota regulate mammalian brain function, opening doors to targeting microbiota for neuroprotective causes.

### 5.1 Limitations of the study

Although our research produced noticeable outcomes, it has several limitations. First, because each group included only a limited number of mice, caution is important when interpreting the results. Second, alterations in gut microbiota composition and function are affected by antibiotic classes, doses, routes, and durations of administration (Lange et al., 2016). A period of 3 weeks was used in this study for the oral administration of antibiotics. No further analysis was performed to determine whether the altered gut microbiota had recovered. It is necessary to perform further research to obtain a deeper understanding of the mechanism.

## Data Availability

The datasets presented in this study can be found in online repositories. The names of the repository/repositories and accession number(s) can be found in the article/Supplementary material.

## Footnotes

^1^ http://www.jsxtsw.com/en/345/

^2^ http://rdp.cme.msu.edu/

^3^ http://www.omicstudio.cn/tool

^4^ http://huttenhower.sph.harvard.edu/LEfSe

^5^ http://www.hmdb.ca/

^6^ https://metlin.scripps.edu/

^7^ http://bioconductor.org/packages/release/bioc/html/ropls.html

^8^ https://cloud.majorbio.com

## Supplementary material

Supplementary Figure S1 Heatmap depicting the expression levels of various metabolites across different groups (BAmox, BCip, and BCon). The color gradient illustrates the relative abundance, with warmer colors indicating higher concentrations. Clustering analysis shows distinct patterns of metabolite profiles among the samples.

SUPPLEMENTARY FIGURE S2 **(A)** Principal component analysis (PCA) diagram of metabolites in all serum samples. **(B)** PLS-DA score chart.

SUPPLEMENTARY FIGURE S3 **(A)** amx vs con; **(B)** cip vs con, KEGG enrichment analysis graph. **(C)** Heat map represent the significantly different metabolites in the groups.

SUPPLEMENTARY FIGURE S4 Hierarchical clustering of correlation between gut microbiota and metabolome downstream.
