# Supplementary figures and images for: Behavioral alterations in antibiotic-treated mice associated with gut microbiota dysbiosis: insights from 16S rRNA and metabolomics
Source: Front Neurosci. 2025 Feb 28;19:1478304. doi: 10.3389/fnins.2025.1478304 (PMC11906700; doi:10.3389/fnins.2025.1478304)

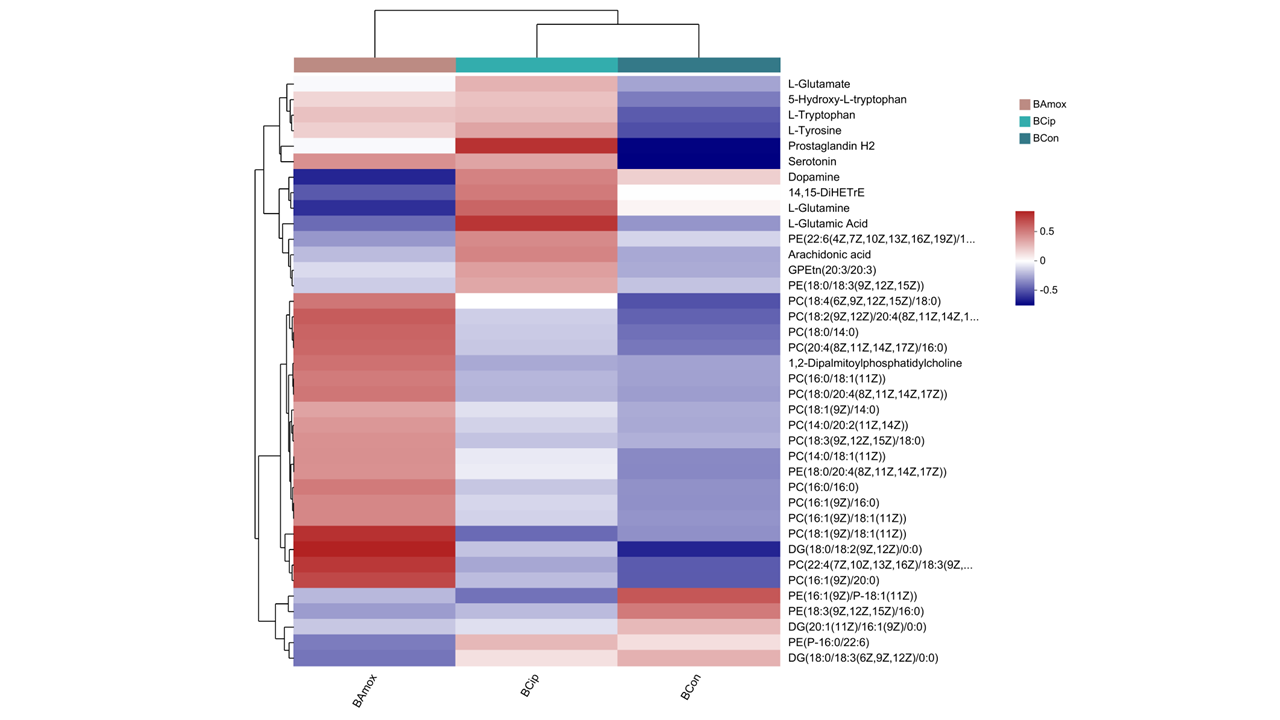

Supplement: Supplementary Figure S1 — Heatmap depicting the expression levels of various metabolites across different groups (BAmox, BCip, and BCon). The color gradient illustrates the relative abundance, with warmer colors indicating higher concentrations. Clustering analysis shows distinct patterns of metabolite profiles among the samples. [file Image_1.tif]

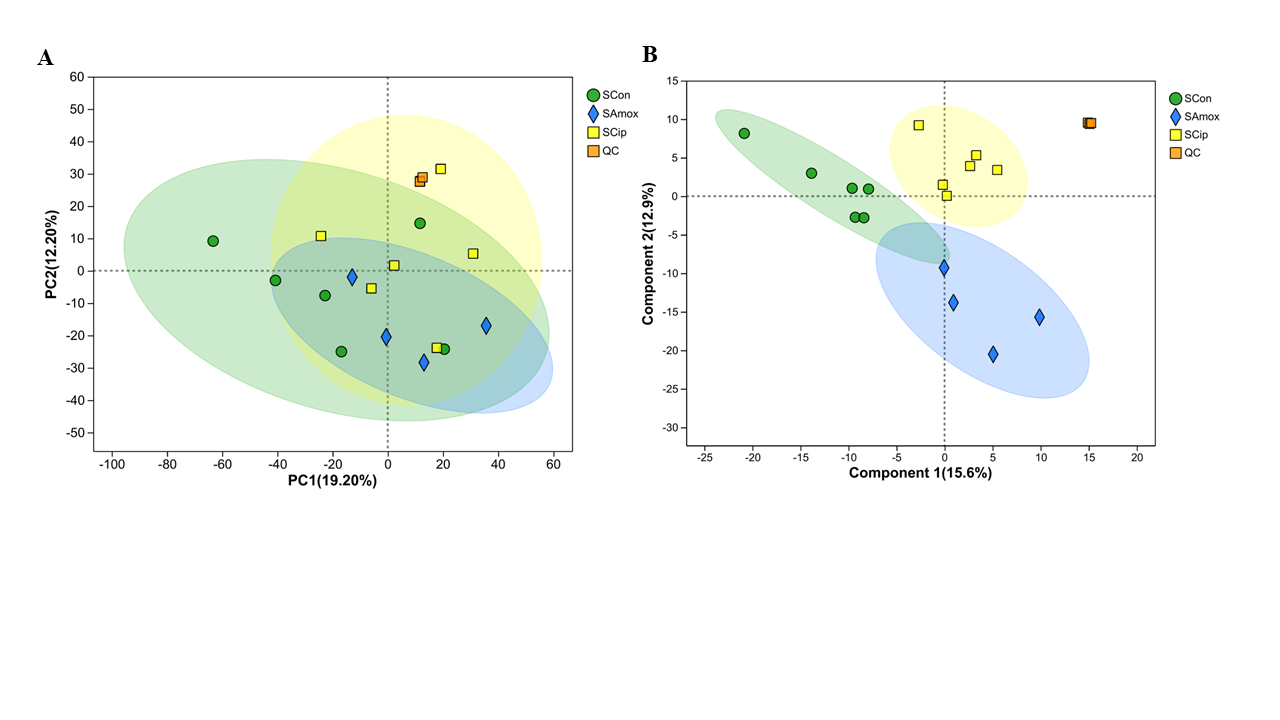

Supplement: SUPPLEMENTARY FIGURE S2 — (A) Principal component analysis (PCA) diagram of metabolites in all serum samples. (B) PLS-DA score chart. [file Image_2.tif]

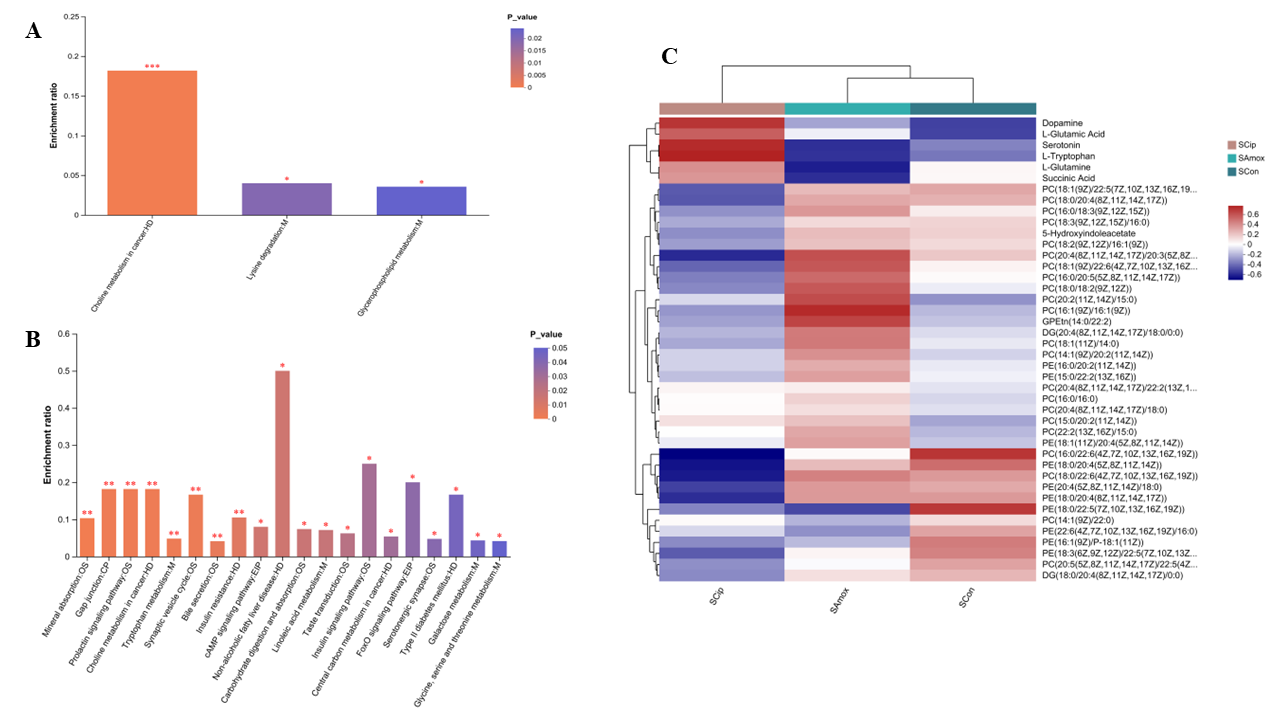

Supplement: SUPPLEMENTARY FIGURE S3 — (A) amx vs con; (B) cip vs con, KEGG enrichment analysis graph. (C) Heat map represent the significantly different metabolites in the groups. [file Image_3.tif]

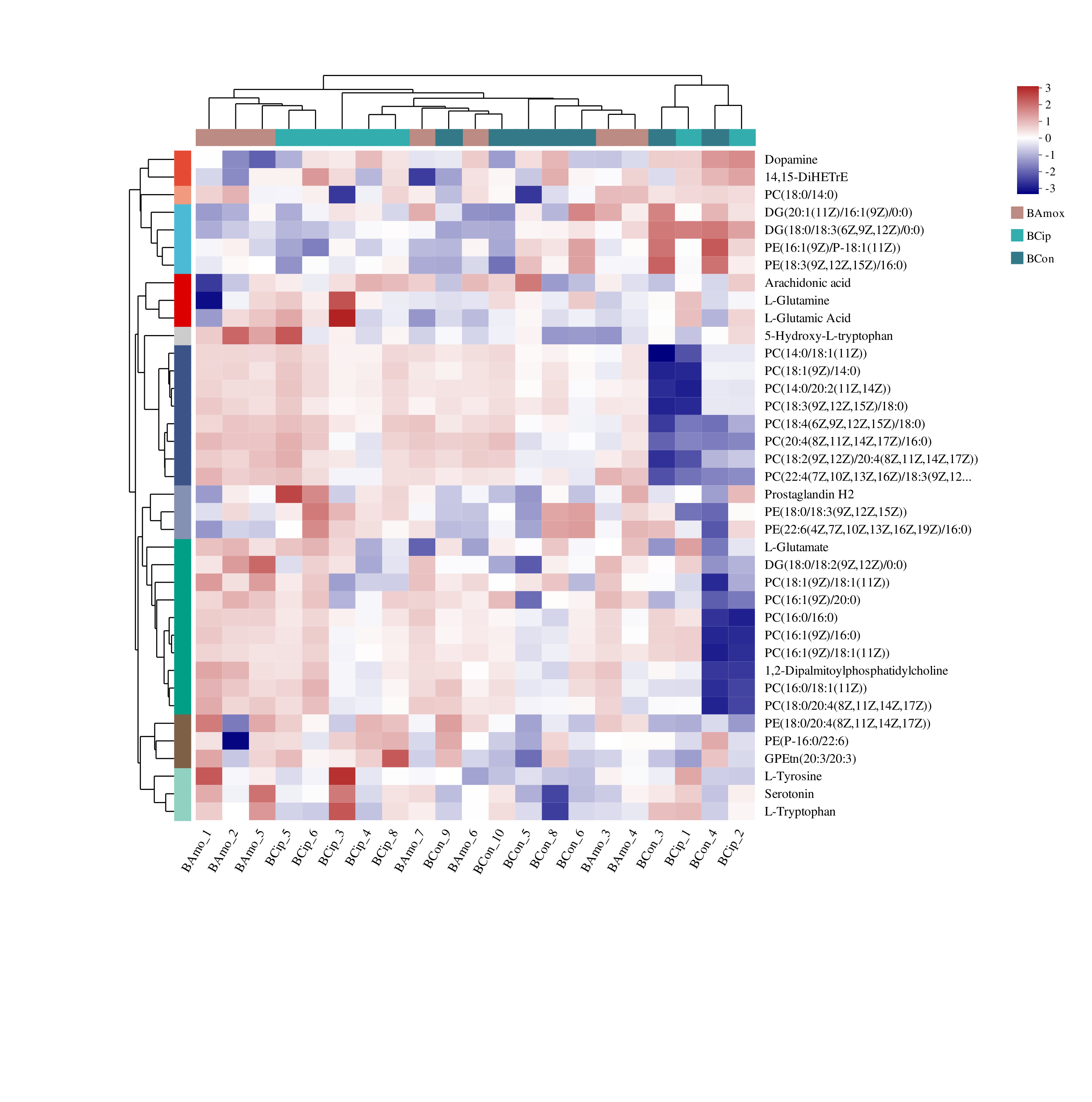

Supplement: SUPPLEMENTARY FIGURE S4 — Hierarchical clustering of correlation between gut microbiota and metabolome downstream. [file Image_4.tiff]
